# The Effect of Extracts and Essential Oil from Invasive *Solidago* spp. and *Fallopia japonica* on Crop-Borne Fungi and Wheat Germination

**DOI:** 10.17113/ftb.58.03.20.6635

**Published:** 2020-09

**Authors:** Sabina Anžlovar, Damjan Janeš, Jasna Dolenc Koce

**Affiliations:** 1 Biotechnical Faculty, University of Ljubljana, Jamnikarjeva 101, 1000 Ljubljana, Slovenia; 2 Faculty of Pharmacy, University of Ljubljana, Aškerčeva 7, 1000 Ljubljana, Slovenia

**Keywords:** antifungal activity, *Fallopia japonica*, essential oil, flower and leaf extracts, *Solidago*, *Triticum aestivum*

## Abstract

**Research background:**

Many plant extracts and essential oils show antibacterial and antifungal activities, with potential to replace the use of synthetic fungicides. We used invasive alien plants goldenrod (*Solidago* spp.) and Japanese knotweed (*Fallopia japonica*) as source materials to determine their antifungal activities against seed-associated fungi from wheat grain (*Alternaria alternata, Alternaria infectoria, Aspergillus flavus, Epicoccum nigrum* and *Fusarium poae*).

**Experimental approach:**

Aqueous and organic extracts (ethanol, methanol and acetone) were prepared from leaves and flowers of *S. canadensis*, *S. gigantea* and *S. virgaurea*, and leaves and rhizomes of *F. japonica*. Additionally, essential oils were distilled from *Solidago* flowers and leaves. The extracts and essential oils were tested as inhibitors of fungal growth *in vitro*. *Solidago* essential oils were tested also as antifungal agents for protection of wheat grain by determining its fungal infection and germination rate.

**Results and conclusions:**

The extracts showed a wide spectrum of low to moderate antifungal activities, with those of *Solidago* spp. generally more effective than those of *F. japonica*, and organic extracts more effective than aqueous extracts. The essential oils from leaves and flowers had similar antifungal activity and whole shoots can be collected for their production. This study presents the systematic study of the composition of essential oils from flowers and leaves of three widely distributed *Solidago* spp. in Slovenia, with the major constituents of terpenes and terpenoids α-pinene, germacrene D and bornyl acetate.

**Novelty and scientific contribution:**

The study presents the first use of *Solidago* spp. and *Fallopia japonica* extracts and essential oils against fungal strains isolated from wheat grain.

## INTRODUCTION

Wheat (*Triticum aestivum* L.) is one of the most important cereal crops, and it is cultivated worldwide. During germination and growth in the field and during storage of the harvested grain, it is under constant attack by damaging microbes, such as seed-borne and soil-borne fungi (*1*). Fungal infections can have negative effects on wheat grain and result in economic losses. In a previous study, we isolated several fungal species from the wheat grain, including *Alternaria alternata*, *Alternaria infectoria*, *Aspergillus flavus*, *Epicoccum nigrum* and *Fusarium poae* (*2*). Among these fungi, *Aspergillus* and *Fusarium* spp. are known to produce harmful mycotoxins that can present serious risks not only for the plants, but also for human and animal health (*3*). Therefore, treatments with fungicides are necessary to protect the plants and/or the harvested crop from spoilage and to reduce the damage. However, the use of synthetic fungicides has led to the development of fungal resistance against antifungal agents and fungicide residues can also be harmful to other organisms (*e.g.* mammals) (*4*). Additionally, the awareness of consumers to use, and hence farmers to produce, organic food is rising. For these reasons, the interest in new and more ‘green’ (*i.e.* more consumer and nature friendly) bioprotective agents has increased.

Plant-derived metabolites are an important source of biologically active compounds (*5*) that can be obtained as extracts and essential oils from a variety of plant species. Among these, there are also invasive alien plant species that can cause problems in new habitats due to their negative impact on the native biodiversity, their effects on human health (*e.g.* allergenic plants), and their associated economic losses (*e.g.* infrastructural damage) (*6*). Regular annual management of such habitats to reduce populations of invasive plants involves organised mowing and/or removal, and therefore large quantities of biomass are readily available. Hence, despite their harmful effects, invasive plants also present a potential source of usable and beneficial compounds. Therefore, in the present study, we tested some invasive members of the *Asteraceae* family, known as goldenrod (*Solidago* spp.), and the *Polygonaceae* family, known as Japanese knotweed (*Fallopia japonica* (Houtt.) Ronse Decr.).

Goldenrod is a herbaceous perennial that mainly originates in North America. In Europe, it is found as the native species of European goldenrod (*Solidago virgaurea* L.), which is used in traditional medicine for infections and inflammation of the bladder and kidney. On the other hand, Canadian goldenrod (*Solidago canadensis* L.) and giant goldenrod (*Solidago gigantea* Aiton) are invasive weeds that form dense populations along roads, railways and other ruderal habitats (*7*). As these have similar properties to *S. virgaurea*, they might also have uses as medicinal plants. Goldenrod contains many biologically active compounds, which include flavonoids, triterpene saponins, diterpene lactones, phenolic glycosides, phenolic acids, tannins and essential oils (*8*). The analyses of these *Solidag*o essential oils in Poland showed high content of terpenes α-pinene, limonene and germacrene D (*9*-*12*). Previous studies of the biological activities of goldenrod had demonstrated mainly antibacterial activities of their extracts (*8*, *13*-*16*) and essential oils (*17*-*19*). On the other hand, their potential antifungal activities have been less studied, although inhibition of fungal growth by goldenrod extracts and essential oils has been reported against *Aspergillus niger* (*16*, *17*), *Candida albicans* (*17*, *20*, *21*), and *Armillaria mellea* and *Fusarium* sp. (*13*).

Japanese knotweed originates in eastern Asia, where it is used in traditional medicine and as food (*22*). In Europe and North America, it is one of the most invasive alien species. It forms dense populations along rivers, roads and railways. Its success in new habitats is primarily related to vegetative reproduction through underground rhizomes, with high regeneration and rapid growth of large shoots that have broad leaves which overgrow and overshadow the nearby plants (*23*). Phytochemical studies of the composition of knotweed rhizomes and shoots have indicated high amounts of quinones, stilbenes, flavonoids, coumarins, lignans and other compounds (*24*). Japanese knotweed is a rich source of resveratrol, which is used as an antioxidant dietary supplement (*25*, *26*). Biological activity for Japanese knotweed has been previously shown, mainly as antibacterial activity of the underground tissues, while its antifungal activity has rarely been reported (*22* and references therein).

Therefore, based on the known biological activities of goldenrod and knotweed and on the availability of a large biomass, we tested the effectiveness of goldenrod and knotweed extracts and goldenrod essential oil against crop-borne pathogenic and saprophytic fungi that the food industry must regularly contend with. In addition, the goldenrod essential oils that showed moderate antifungal activities were tested for effects on germination of wheat grain and on early growth of seedlings.

## MATERIALS AND METHODS

### Plant material

Goldenrod (*Solidago* spp.) was collected in the flowering period in August from various locations. Fresh shoots of *S. virgaurea* were collected at four locations in Zasavje, Slovenia (N 46°7'12.80'', E 15°6'20.50''; N 46°6'28.00'', E 15°7'15.96''; N 46°9'1.08'', E 14˚56'41.82''; and N 46°8'58.13'', E 14°55'40.39''), fresh shoots of *S. canadensis* were collected in Ljubljana, Slovenia (N 46°4'19.86'', E 14°25'52.25''), and fresh shoots of *S. gigantea* were collected at two locations in Ljubljana, Slovenia (N 46°3'29.44'', E 14°27'50.30''; and N 46°3'17.61'', E 14°27'11.34''). The leaves and flowers were separately removed from the shoots and air dried at room temperature in the dark.

Mature leaves and rhizomes of *Fallopia japonica* (Houtt.) Ronse Decr. were collected in June and November, respectively, in a dense stand next to the stream Mali Graben, in Ljubljana, Slovenia (N 46°02'33.9'', E 14°27'00.9''). The fresh leaves were separated from the shoots and dried at 60 °C in a forced ventilation oven (Heraeus Instruments, Hanau, Germany). Fresh rhizomes were washed with tap water, cut into 1-cm thick pieces, frozen in liquid nitrogen, lyophilised (Alpha 1-4LSC; Christ, Osterode am Harz, Germany), and stored at room temperature in the dark.

### Preparation of aqueous and organic extracts

The extracts were prepared as previously described (*13*). Dry *Solidago* spp. leaves and flowers were ground in a mill (M20; IKA-Werke, Staufen, Germany), and 50 g of this ground material were dissolved in 300 mL distilled water for the aqueous extract, or in 300 mL 96% ethanol for the organic extract. The mixtures were left shaking at 175 rpm for 24 h at room temperature on an orbital shaker (Laboshake 500; Gerhardt, Königswinter, Germany). After the extraction, the mixtures were vacuum filtered (520A filter paper; Whatman, GE Healthcare Life Sciences, Maidstone, UK) and the solvents were evaporated under reduced pressure at 40 °C using a rotary flask evaporator (Rotavapor R-124, Vacobox B-177, Waterbath B-480; Büchi, Flavil, Switzerland). These dry crude extracts were stored in sterile glass bottles at room temperature in the dark.

Dry *F. japonica* leaves and rhizomes were ground in a mill (M20; IKA-Werke), and 50 g ground material were dissolved in 600 mL distilled water for the aqueous extract, or in 300 mL absolute acetone, methanol or ethanol for the different organic extracts. Dry plant material absorbed more water than organic solvents, therefore higher volume was needed for the aqueous extracts. The mixtures were then processed to the crude extracts as for the *Solidago* spp. (see above) and stored in sterile glass bottles at room temperature in the dark.

To determine the antifungal activities of these extracts from *Solidago* spp. and *F. japonica*, 500 mg of crude extract were resuspended in 1 mL distilled water or 1 mL ethanol (for aqueous and organic extracts, respectively). The extracts (*γ*_final_=500 mg/mL) were vortexed and stored at 4 °C until added onto the surface of the fungal growth medium.

### Distillation and gas chromatography–mass spectrometry analysis of goldenrod essential oil

A mixture of 4 L deionised water with either 200 g dry *Solidago* spp. flowers or 300 g dry *Solidago* spp. leaves was placed in a 5-litre round-bottomed flask, and connected to a Clevenger apparatus. The heating was regulated to generate a boiling and condensation speed of 2-3 mL/min. The boiling time was 4 h. Following complete distillation, the essential oils were collected and the volume measured in a graduated tube of the Clevenger apparatus. Then, the essential oils were carefully separated from the water phase. Yield of essential oil (mL/kg) was calculated as a ratio between the measured volume of essential oil and the mass of dry plant material used for the distillation. Solutions of essential oil were prepared in hexane (*φ*=0.005; gas chromatography grade; Merck, Darmstadt, Germany) for gas chromatography–mass spectrometry analysis.

A gas chromatograph (GCMS-QP2010 Ultra; Shimadzu, Kyoto, Japan) was used to analyse the material. A fused silica column was used (Rxi-5 Sil MS; 30 m×0.25 mm i.d.; film thickness, 0.25 μm; Restek, Bellefonte, PA, USA). The temperature programme began at 50 °C for 5 min, and was then raised to 250 °C at 3 °C/min, and then held at 250 °C for 5 min. The injection temperature was 250 °C, the temperature of the ion source was 200 °C, and the temperature of the interface was 270 °C. The injection volume was 1 µL, the split ratio 1:100, the carrier gas He, and the flow 1 mL/min linear velocity. The mass spectrometry conditions included electron impact mode at an ionisation voltage of 70 eV, with total ion current recorded, and a scan range from 35 to 500 *m/z* at a frequency of 5 Hz. The detector voltage was 1 kV. The total analysis time was 76.7 min.

The compounds were first identified by comparing their mass spectra and retention indices to the spectra and retention indices from the Flavors and Fragrances of Natural and Synthetic Compounds spectral library (*27*) and National Institute of Standards and Technology spectral library (*28*). Their identities were then confirmed by comparing them with mass spectra and retention indices of the reference compounds from Sigma-Aldrich, Merck (Steinheim, Germany) for the following compounds: bornyl acetate, caryophyllene oxide, *p*-cymene, limonene, myrcene, α-pinene, and β-pinene. Retention indices were calculated logarithmically with reference to *n*-alkanes as described previously (*9*, *11*). Concentrations were calculated as relative peak areas.

### Antifungal activities

The antifungal activities of the *Solidago* spp. and *F. japonica* extracts and the *Solidago* spp. essential oils were tested against five moulds: *A. alternata*, *A. infectoria*, *A. flavus, E. nigrum* and *F. poae*. These fungi were previously isolated from wheat grain and identified by molecular methods (*2*). The majority of these fungal isolates are saprophytic, and *A. flavus* and *F. poae* are considered to be plant pathogens.

The inhibitory effects of the *Solidago* spp. and *F. japonica* extracts on radial growth of the fungal mycelia was tested following the method described in our previous study (*13*). A volume of 50 μL of aqueous or organic extract (*γ*=500 mg/mL) was spread over each Petri dish (*d*=90 mm) containing potato dextrose agar (0.02 mass per volume ratio; Biolife, Milano, Italy) using a Drigalski spatula. Disks of fungal mycelia (*d*=5 mm) were cut from the margins of 7-day-old stock cultures and aseptically inoculated by placing them in the centre of the medium with the extract. Control samples without extracts (for aqueous extracts) and with ethanol (for organic extracts) were prepared at the same time. Fungicide azoxystrobin (10 mg/mL) was used as the standard reference for antifungal activity. The fungal colonies were incubated at room temperature ((23±2) °C) in the dark for 7 days.

The inhibitory effects of the *Solidago* spp. essential oils on radial growth of the fungal mycelia were tested using the agar dilution method, similar to that described by Zabka *et al.* (*29*) and in our previous studies (*2*, *30*). The pure essential oils were added to the autoclaved potato dextrose agar medium (Biolife) at approx. 40 °C to prepare the final volume fractions of essential oils *φ*=0.001, 0.0005 and 0.0001. The medium was poured in the Petri dish (*d*=90 mm) and left to solidify. Control samples without essential oil were prepared at the same time. Disks of fungal mycelia (*d*=5 mm) were cut from the margins of 7-day-old stock cultures and aseptically inoculated by placing them in the centre of the medium. Petri dishes were sealed with parafilm to prevent evaporation of the essential oil. The fungal colonies were incubated at room temperature ((23±2) °C) in the dark for 7 days.

Inhibition of the fungal radial growth was calculated according to Lira-De León *et al.* (*5*):

Inhibition=((*d*(control colonies)-*d*(treated colonies))/*d*(control colonies)·100 /1/

where *d* is the mean diameter of the fungal colony. Three replicates were carried out for each treatment.

### Germination and fungal contamination of wheat grain

Wheat (*Triticum aestivum*) grain from the Vila Natura ecological farm (Vučja vas, Prlekija, Slovenia) was used to evaluate the fungicidal activities of the *Solidago* spp. essential oils. Non-sterile wheat grain (10 g) was treated with *Solidago* spp. essential oils (*φ*=0.02) prepared by dilution of the concentrated essential oils in dimethylsulphoxide (*φ*=0.1), as described by Anžlovar *et al.* (*2*). The grains were submerged and incubated in the essential oils in Petri dishes sealed with parafilm and under shaking (50 rpm) at (25±2) °C for 24 h. The control grains were soaked in dimethylsulphoxide (*φ*=0.1), also with shaking.

After the treatments with the essential oils, fungal infection and seed germination were quantified using the direct plating method (*2*). Following the treatments, 10 subsamples of 10 wheat grains were placed on potato dextrose agar (0.02 mass per volume ratio) in Petri dishes (*d*=90 mm) and incubated at (25±2) °C in the dark. The development of fungal colonies on the surface of the wheat grain was monitored daily, and after 74 h the fungal infections were quantified by counting the number of infected grains, expressed as a percentage of the total used grains. At the same time, the germination of the wheat grain was examined by counting the number of germinated grains, again expressed as a percentage of the total used grains.

### Statistical analysis

For the antifungal activities, three fungal colonies per treatment were measured and mean values were calculated. From these data, inhibition of fungal growth was calculated. Mean values and standard errors were calculated for the fungal infection and germination of the wheat grain treated with essential oils (*N*=10 per treatment).

The significance (p<0.05) of the differences between the treated and control samples in antifungal and germination experiments was tested using one-way ANOVA and Tukey’s *post-hoc* tests. Statistical analysis was performed using the Prism v. 5.01 software (*31*).

## RESULTS AND DISCUSSION

This study was conducted to evaluate the potential of extracts from the invasive plants goldenrod and knotweed, and the essential oils from goldenrod, as antifungal and preservative agents against five fungal species that were previously isolated from wheat grain (*2*), each with the potential for crop spoilage. These fungal species inhabit different ecological niches where they function as saprophytes and/or pathogens: *A. alternate* and *A. infectoria* as endophytes/ saprophytes, *A. flavus* as a saprophyte/ pathogen, *E. nigrum* as a saprophyte, and *F. poae* as a pathogen. *Solidago* spp. and *F. japonica* extracts and *Solidago* spp. essential oils were prepared separately from the leaves, rhizomes and/or flowers and were spread over the fungal growth medium. The data show the greatest antifungal activities for the *Solidago* spp. essential oils, although the *Solidago* spp. leaf and flower extracts and the *F. japonica* rhizome extracts also have promising potential for antifungal treatments.

### Composition of the Solidago spp. essential oils

Data on the yields of the *Solidago* spp. essential oils and their chemical composition (Table 1) show that *S. canadensis* yielded higher amounts of essential oils than *S. gigantea* and *S. virgaurea*, and for all three of these species the essential oils yield from flowers was higher than from leaves. Similarly, a higher amount of essential oils was found in the flowers than in other aerial parts of *S. canadensis* (*32*). The essential oil of *S. virgaurea* had the highest number of tentatively identified compounds.

**Table 1:** Composition of flower and leaf essential oils from *S. canadensis*, *S. gigantea* and *S. virgaurea*. For each of the essential oils, the five compounds showing the highest levels for the relative peak areas are given in italic

Compound	Identification	RI		Relative peak area for essential oil/%											
		*S. canadensis*				*S. gigantea*				*S. virgaurea*			
Database	Measured	Flower			Leaf	Flower		Leaf		Flower		Leaf	
*Terpenes and terpenoids*															
α-Pinene	MS, RC, RI	933	931	*33.1*			2.7	*7.6*		1.8		*28.5*		*13.1*	
β-Pinene	MS, RC, RI	978	974	*2.6*			0.7	1.0		0.4		4.6		2.7	
Myrcene	MS, RC, RI	991	988	1.0			0.2	0.7		0.6		*5.1*		2.3	
Limonene	MS, RC, RI	1030	1027	*21.5*			*2.8*	1.0		0.8		3.6		2.8	
Bornyl acetate	MS, RC, RI	1285	1282	*3.8*			*5.5*	*4.1*		*9.1*		1.2		1.5	
α-Gurjunene	MS, RI	1406	1406	0.8			*2.9*	*4.4*		nd		0.1		3.0	
α-Humulene	MS, RI	1454	1454	0.4			0.4	0.4		0.4		*4.9*		*4.3*	
γ-Muurolene	MS, RI	1478	1474	nd			0.2	1.3		*2.0*		0.3		0.3	
Germacrene D	MS, RI	1480	1479	*10.4*			*48.7*	*20.8*		*33.8*		*8.4*		6.3	
δ-Cadinene	MS, RI	1518	1517	0.3			0.4	1.5		*2.3*		1.6		1.5	
Spathulenol	MS, RI	1576	1574	1.4			2.0	2.3		*2.4*		2.0		*6.5*	
Caryophyllene oxide	MS, RC, RI	1587	1579	1.1			1.2	1.9		1.2		*4.8*		*10.2*	
Humulene epoxide II	MS, RI	1613	1607	0.3			0.4	0.6		0.5		3.1		*6.7*	
Cyclocolorenone	MS, RI	1757	1749	0.6			*7.6*	3.3		nd		nd		nd	
*Aromatic compounds*															
*p*-Cymene	MS, RC, RI	1025	1022	0.9			0.1	*3.6*		0.2		0.2		0.3	
*Y*(essential oil)/(mL/kg)					8.3	5.5			2.0		1.2		1.8		1.0	

MS=mass spectrum, RC=reference compound, RI=retention index; *Y*=yield, nd=not detected

**Fig. S1:**
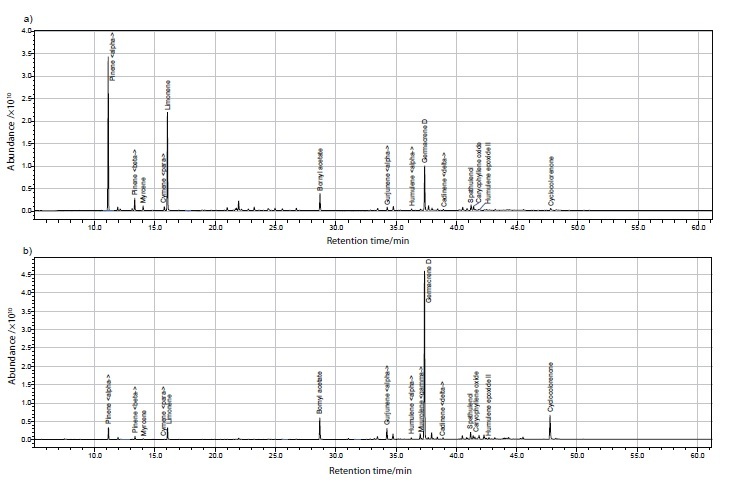
GC-MS chromatogram of essential oil from *S. canadensis*: a) flowers and b) leaves

In all of the samples, terpenes and terpenoids were the major components, although the compositions differed according to the *Solidago* spp. and the plant tissue used for the preparation of the essential oils (Table 1). The data available in the literature generally relate to the composition of *S. canadensis* essential oils. The major components of essential oils from *S. canadensis* shoots and flowers have been reported as α-pinene and germacrene D (*12*), and as α-pinene, germacrene D and (*E*)-nerolidol (*33*). For *S. canadensis* flowers more specifically, the major constituents of the essential oils have been reported as α-pinene, γ-cadinene and limonene (*10*) and as α-pinene, germacrene D and curlone (*34*). The main constituent of essential oils from *S. canadensis* roots is thymol (*17*). In the present study, the major essential oils from *S. canadensis* flowers were α-pinene, limonene, germacrene D, bornyl acetate and β-pinene, while those from *S. canadensis* leaves were germacrene D, cyclocolorenone, bornyl acetate, α-gurjunene and limonene (Table 1 and Fig. S1), which are generally comparable to the data published by Kalemba *et al.* (*10*, *12*).

**Fig. S2:**
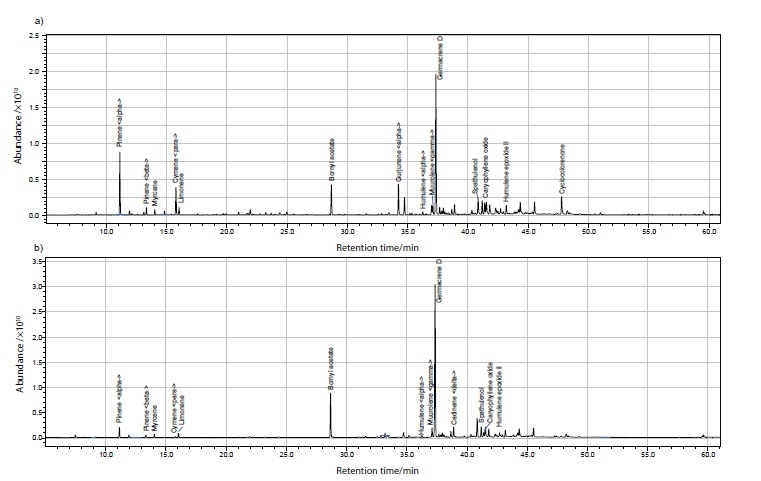
GC-MS chromatogram of essential oil from *S. gigantea*: a) flowers and b) leaves

**Fig. S3:**
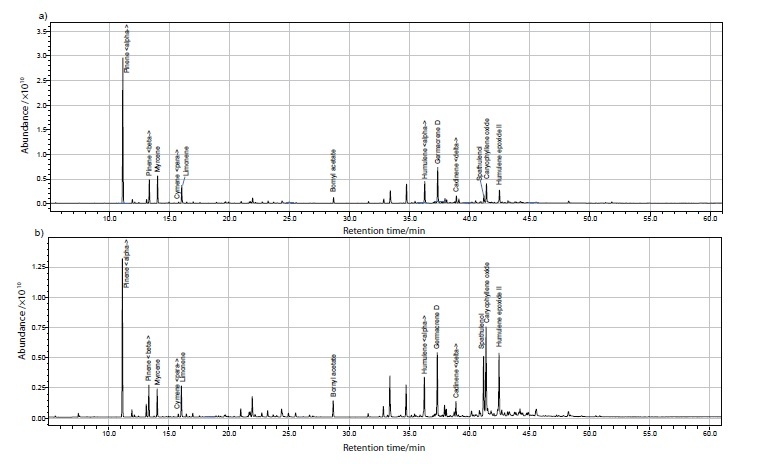
GC-MS chromatogram of essential oil from *S. virgaurea*: a) flowers and b) leaves

The *S. gigantea* flowers yielded the five main essential oils of germacrene D, α-pinene, α-gurjunene, bornyl acetate and *p*-cymene, while for the leaves these were germacrene D, bornyl acetate, spathulenol, khusinol and δ-cadinene (Table 1 and Fig. S2). This composition is similar to the results of Kalemba *et al. (**11*) and Kalemba and Thiem (*12*) who reported also the content of cyclocolorenone up to 35%. In our study this content was lower, around 3% in the flower essential oil while in leaves it was not detected. The essential oil of native species *S. virgaurea* (Table 1 and Fig. S3) contained α- and β-pinene, myrcene and caryophyllene oxide which is similar to the studies of Kalemba *et al. (**9*), and α-humulene and germacrene D as reported by Kalemba and Thiem (*12*). The five major components in *S. virgaurea* flower essential oils were α-pinene, germacrene D, myrcene, α-humulene and caryophyllene oxide, while for the leaves these were α-pinene, caryophyllene oxide, humulene epoxide II, spathulenol and germacrene D.

A comparison of the essential oil compositions across all of these samples shows that germacrene D is the most frequently found as one of the top five components, followed by α-pinene and bornyl acetate. *S. virgaurea* stands out here too, with high proportions of caryophyllene oxide, humulene epoxide, spathulenol and α-humulene in both the flower and leaf essential oils, with myrcene also in the flower essential oils, along with some compounds that were not found in the other *Solidago* spp.: benzoates, benzyl salicylate and ketones. The *S. canadensis* flower and leaf essential oils and the *S. gigantea* flower essential oils had relatively high proportions of limonene, cyclocolorenone and *p*-cymene, respectively. The *S. canadensis* leaf essential oils and the *S. gigantea* flower essential oils stand out too, as they both showed relatively high proportions of α-gurjunene. Grul'ová *et al.* (*35*) report about remarkable variations in the chemical composition of essential oils of invasive goldenrod populations among collection areas both within the same species (*S. canadensis*) and between the two species (*S. canadensis* and *S. gigantea*).These results and previous studies combined thus indicate that the composition of *Solidago* essential oils is highly variable. However, in most cases, direct comparisons should be made with care, as the essential oils were not always distilled from the flowers and leaves separately.

### Antifungal activities of the extracts and essential oils

The antifungal activities of the *Solidago* spp. and *F. japonica* extracts were tested using the spread plate method against the five fungal species. These fungi were differentially susceptible to the plant extracts, although in general the *Solidago* spp. extracts were more effective than the *F. japonica* extracts, and the organic extracts were more effective than the aqueous extracts.

Overall, the extracts from all three *Solidago* spp. showed similar inhibition of fungal growth (22-25%), with no significant differences among the species (p=0.8775). The extracts of *S. canadensis* provided a maximum of 61.8% inhibition, *S. gigantea* of 47.1%, and *S. virgaurea* of 56.9%, with all of these maxima obtained against *F. poae* (Table 2). Between the extracts in terms of the two plant tissues (*i.e.*, leaves, flowers) and in terms of the two solvents (*i.e*., water, ethanol), there were no significant differences for this inhibition of fungal growth (p=0.6394; p=0.1769; respectively).

**Table 2:** Antifungal activities of the leaf and flower aqueous and ethanolic extracts of the *Solidago* spp. The inhibition of fungal growth was calculated from the mean (*N*=3) diameters of the fungal colonies for the control and extracts

Source	Extract	Growth inhibition/%				
*A. alternata*	*A. infectoria*	*A. flavus*	*E. nigrum*	*F. poae*
*S. canadensis*						
Leaf	Aqueous	50.08	-0.27	22.20	33.63	39.82
	Ethanol	26.42	13.96	6.38	22.20	55.25
Flower	Aqueous	37.24	-0.07	11.14	19.72	29.80
	Ethanol	27.43	7.54	3.53	29.85	61.76
*S. gigantea*						
Leaf	Aqueous	17.94	-0.95	16.68	19.28	40.75
	Ethanol	38.53	9.24	7.62	46.43	47.12
Flower	Aqueous	20.15	0.33	5.89	42.45	22.40
	Ethanol	37.81	4.64	2.11	40.46	29.91
*S. virgaurea*						
Leaf	Aqueous	12.21	3.30	9.45	34.31	17.17
	Ethanol	33.00	9.72	13.88	32.38	55.75
Flower	Aqueous	8.83	1.12	11.15	48.61	33.59
	Ethanol	25.66	6.61	5.58	29.90	56.94
Azoxystrobin		43.32	47.12	94.75	63.50	69.24

Negative numbers indicate growth stimulation

The susceptibilities of the fungal species to the *Solidago* spp. extracts from the most to the least were: *F. poae* (mean growth inhibition calculated from the data in Table 2 (40.9±14.7) %)>*E. nigrum* ((33.3±9.9) %)>*A. alternata* ((27.9±12.0) %)*>A. flavus* ((9.6±5.8) %)>*A. infectoria* ((4.6±4.8) %). These data show that the leaves and/or flowers of all three *Solidago* spp. could indeed be used as antifungal treatments. These data also indicate the wide range of fungal growth inhibition obtained for these *Solidago* spp. extracts. Webster *et al.* (*36*) tested an aqueous extract of *S. gigantea* and also reported moderate antifungal activity against some pathogens and moulds, but not against *A. flavus*.

Higher antifungal activities might instead be predicted for extracts from the underground plant tissues (*i.e*. roots), because these generally contain higher amounts of biologically active compounds such as rutin, catechin, epicatechin, and chlorogenic and ferulic acids, and others (*13*). In the present study, we used the above-ground shoots because they are regularly harvested to maintain the infrastructure and to control invasive plants, thus potentially providing a large biomass.

*Fallopia japonica* is less aromatic than these *Solidago* spp., although it is a well-known and rich source of biologically active compounds (*e.g.* resveratrol, catechin, and others), with higher amounts reported for the underground tissues (*22*, *26*). It is a very invasive species, and one of the best ways to eradicate the plants and to control their distribution is regular mechanical cutting of the shoots and/or digging out the below-ground parts combined with chemical treatments (*37*). For this reason, we prepared *F. japonica* extracts from leaves and rhizomes separately in water and in different organic solvents. The data on these antifungal activities show that the rhizome extracts inhibited fungal growth more effectively than the leaf extracts (p=0.0182) (Table 3). Indeed, the leaf extracts not only had lower antifungal activities, but they also even stimulated the growth of *A. alternata, E. nigrum* and *F. poae*. This beneficial effect might be related to fungal hormesis, which is when fungi positively react to low concentrations of specific compounds that stimulate their growth (*38*). On the other hand, the choice of extraction solvent did not significantly affect the antifungal activities of these *F. japonica* extracts (p=0.2982). Here, the ethanol and methanol extracts were generally the most effective, while being similar, followed by the acetone extracts, with the aqueous extracts as the least effective. The higher activities of the ethanol extracts might be related to the higher content of phenolic compounds, which are extracted better in polar solvents like ethanol and methanol (*39*).

**Table 3:** Antifungal activities of the aqueous and organic leaf and rhizome extracts of *F. japonica*. The inhibition of the fungal growth was calculated from the mean (*N*=3) diameters of the fungal colonies for the control and extracts

Source	Extract	Growth inhibition/%				
*A. alternata*	*A. infectoria*	*A. flavus*	*E. nigrum*	*F. poae*
Leaf	Aqueous	-29.46	1.15	82.15	-15.57	-59.09
	Acetone	-29.17	19.62	17.83	-27.10	-103.96
	Methanol	-46.93	23.19	11.72	-35.59	-71.14
	Ethanol	-56.83	37.43	16.08	-34.51	61.56
Rhizome	Aqueous	-0.19	-9.27	-7.99	25.11	-49.61
	Acetone	27.52	19.12	7.26	54.02	-10.85
	Methanol	41.23	58.18	8.85	76.83	55.22
	Ethanol	36.61	69.60	22.18	71.96	59.73

Negative numbers indicate growth stimulation

The susceptibilities of the fungal species to the most effective *F. japonica* extracts (*i.e.* rhizome with methanol and ethanol) were as follows, from most susceptible to least (calculated from the data in Table 3): *E. nigrum* ((74.4±3.4) %)>*A. infectoria* ((63.9±8.1) %)>*F. poae* ((57.5±3.2) %)>*A. alternata* ((38.9±3.3) %)*>A. flavus* ((15.5± 9.4) %). When all of the data on the growth inhibition are taken into account, the means are lower and the order of the fungal susceptibilities is different: *A. infectoria*>*A. flavus*>*E. nigrum*>*A. alternata*>*F. poae.* Although antifungal activities of *F. japonica* extracts have rarely been reported, Peng *et al.* (*22*) defined inhibitory effects for *Trichophyton rubrum*, *Microsporum gypseum*, *Fonsecaea pedrosi* and *Candida albicans*.

In addition to these *Solidago* spp. and *F. japonica* extracts, the antifungal activities were also investigated for the *Solidago* spp. essential oils against the same five fungal species, and here three different volume fractions were used to determine whether these activities are concentration dependent. The inhibition of fungal growth by essential oils and extracts can not be directly compared due to different extraction methods and concentrations but generally the essential oils have higher activty than extracts as shown here and by others (*40*). The highest volume fraction (*φ=*0.001) of *Solidago* spp. essential oils generally showed the greatest inhibition of fungal growth across these treatments. However, for *A. infectoria* treated with *S. canadensis* leaf essential oils and *S. virgaurea* flower essential oils, and *E. nigrum* and *F. poae* treated with *S. gigantea* flower essential oils, the lower volume fraction of *φ=*0.0005 essential oils showed greater inhibition than *φ=*0.001 (Table 4).

**Table 4:** Antifungal activities of *Solidago* spp. essential oils prepared from the three species and for two tissues. The inhibition of the fungal growth was calculated from the mean (*N*=3) diameters of the fungal colonies for the control and extracts

Essential oil source	*φ*(essential oil)	Growth inhibition/%				
*A. alternata*	*A. infectoria*	*A. flavus*	*E. nigrum*	*F. poae*
*S. canadensis*						
Leaf	0.0010	39.1	39.8	45.3	68.3	56.7
	0.0005	31.1	52.2	21.6	59.6	54.4
	0.0001	13.0	23.0	8.0	17.0	48.0
Flower	0.0010	39.1	66.1	35.5	66.6	57.9
	0.0005	28.9	34.3	19.6	43.9	50.9
	0.0001	0.0	23.0	3.0	3.0	40.0
*S. gigantea*						
Leaf	0.0010	48.6	69.9	49.2	74.2	64.6
	0.0005	42.2	64.2	41.2	64.9	59.6
	0.0001	20.0	34.0	8.0	63.0	44.0
Flower	0.0010	50.0	77.4	47.2	67.7	58.4
	0.0005	40.0	64.2	43.1	68.4	59.6
	0.0001	20.0	24.0	3.0	56.0	41.0
*S. virgaurea*						
Leaf	0.0010	55.8	78.5	55.8	76.0	74.2
	0.0005	40.0	59.7	40.0	63.2	68.4
	0.0001	18.0	22.0	18.0	49.0	51.0
Flower	0.0010	38.4	53.8	47.9	68.3	55.6
	0.0005	28.9	63.4	28.9	52.6	49.1
	0.0001	0.0	18.0	10.0	14.0	49.0

The essential oils of *S. virgaurea* and *S. gigantea* showed greater inhibition (calculated from the data in Table 4; (60.8±13.1) and (60.7±11.5) %, respectively) than those of *S. canadensis* ((51.4±13.0) %), although this difference did not reach statistical significance (p=0.1928). The antifungal activities were similar when essential oils from leaves and flowers were used (p=0.3598), which is in agreement with the similar chemical compositions of these tested essential oils (Table 1). The susceptibility of the fungal species to the essential oils were as follows, from most susceptible to least: *E. nigrum* ((70.2±3.9) %)>*A. infectoria* ((64.3±15.0) %)>*F. poae* ((61.2±7.08) %)>*A. flavus* ((46.8±6.6) %)>*A. alternata* ((45.2±7.3) %). *Solidago* spp. essential oils have been tested previously mainly in terms of their antibacterial activities, while antifungal treatments are rare, and different effects have been reported. Essential oils from *S. canadensis* roots showed mild to moderate inhibition of growth of *A. niger* and *C. albicans*, respectively (*17*), while essential oils from *S. canadensis* leaves did not inhibit human pathogenic fungi (*18*) but showed promising antifungal activity against *M. fructicola* at mass concetration 1000 g/mL which was significantly higher than of Azoxystrobin (*φ*=0.001). On the other hand only the moderate activity was observed against *A. niger* and no activity against *B. cinerea* (*41*). In the present study, however, we show concentration-dependent inhibitory effects for the leaf and flower essential oils on all five of these seed-associated fungi. For this reason, whole *Solidago* spp. shoots can be used to produce these essential oils, without the need to separate leaves and flowers. According to the composition of essential oils and the results of antifungal activity it is most likely that the major components germacrene D, α-pinene and bornyl acetate are also the most responsible for the reduction of fungal growth. However, other compounds even in smaller amounts can affect growth too due to the sinergistic effects (*40*, *42*). The same wheat-borne fungi were used in our previous study with thyme essential oil (*2*). The comparision of antifungal activities of goldenrod and thyme essential oils shows that *Solidago* oil has lower antifungal activity than thyme. Nevertheless, the biomass of goldenrod is much higher than of thyme therefore further studies of more effective prepararions should be continued, also with other harmful organisms.

The treatments with these essential oils also resulted in bleaching of *A. flavus* mycelia, as these changed colour from green in controls to yellow, and even white, when the highest concentrations of the essential oils were applied (data not shown). Such colour changes for the mycelia might correspond to reduced virulence (*43*).

### Fungal infection and germination of wheat grain

Currently, natural protective agents are getting more attention due to their biodegradability and lower toxicity for humans and the environment, compared to synthetic agents. The presence and growth of fungi in and on cereal grain can have devastating effects on the quality and quantity of a harvest, and some fungal contaminations present danger for human health due to their production of mycotoxins. Therefore, the positive results in these analyses of the antifungal activities of the *Solidago* spp. essential oils can be applied to their use as potential fungicidal agents or preservatives, as has been shown for *S. canadensis* essential oils and postharvest strawberry preservation against *Botrytis cinerea* (*44*).

**Fig. 1:**
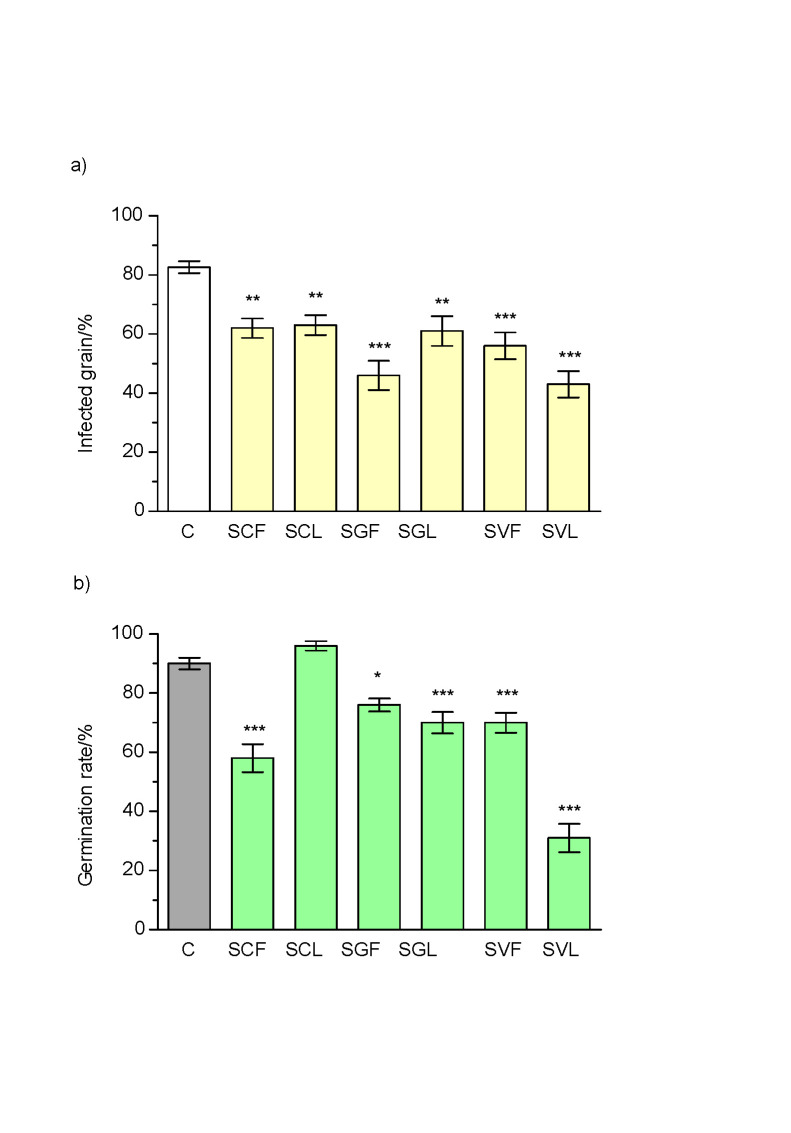
Fungal infection (a) and germination rates (b) of the wheat grain 3 days after the treatments with *Solidago* spp. essential oils (*φ*=0.02). C=control, SCF=*S. canadensis* flowers, SCL=*S. canadensis* leaves, SGF=S. *gigantea* flowers, SGL=*S. gigantea* leaves, SVF=*S. virgaurea* flowers, SVL=*S. virgaurea* leaves. Data present mean values±S_N_ (*N*=3); S_N_=uncorrected sample standard deviation. Statistical analysis: *p<0.05, **p<0.01, ***p<0.001

In the present study, the potential of these *Solidago* spp. essential oils for antifungal treatments of wheat grain was tested. The fungal infections of the grain and the germination rates were monitored for these unsterilised wheat grain, using the same material that was used for previous isolation of fungi. After the treatment with essential oils (*φ=*0.02), the grains were monitored for 3 days. The first fungal infections were observed one day following germination, although only in the grain with the control treatment. On the second day, fungal infections also occurred on 28 to 59% of the treated grain (data not shown). These infections further increased on the third day, to 43 to 63%. Nevertheless, the treatments of the wheat grain with these *Solidago* spp. essential oils significantly reduced the fungal infections compared to the untreated controls, with infection rates for the controls of up to 83% (Fig. 1a).

After 3 days, the lowest fungal infections were observed for the treatments with the *S. gigantea* essential oils ((44.5±14.7) %), followed by *S. virgaurea* ((58.5±15.0) %) and *S. canadensis* ((62.5±10.2) %). These differences were significant only for the *S. gigantea* essential oils (p=0.005 *vs. S. virgaurea*; p<0.0001 *vs. S. canadensis*). On the other hand, the differences among these essential oils due to the tissues used (*i.e.* leaves, flowers) did not show significance (p=0.8032).

In addition, the germination rates of these wheat grain were monitored for 3 days, to determine the influence of the *Solidago* spp. essential oils on seed germination and the early growth of the seedlings (Fig. 1b). All of these *Solidago* spp. essential oils significantly decreased wheat germination, except for those from the leaves of *S. canadensis*. For *S. virgaurea* and *S. gigantea*, the essential oils from leaves inhibited germination more than those from flowers (p<0.0001and p=0.1769, respectively). On average, the highest germination rates were seen for wheat grain treated with *S. canadensis* essential oils ((77.0±22.3) %), followed by *S. gigantea* ((73.0±9.8) %) and *S. virgaurea* ((50.5±23.7) %).

Phytotoxic effects of essential oils are well known, and have been reported previously for many plant species, including *S. canadensis* (*45*). Essential oils have also been proposed as allelopathic bioherbicides because they effectively inhibit the germination of weed seeds (*46*-*48*). In the present study, all of these essential oils except for those from leaves of *S. canadensis* significantly reduced germination rates of these wheat grain. In combination with the data on the fungal contamination, we can conclude that these *Solidago* spp. essential oils have the potential to preserve the wheat grain after harvest. On the other hand, the comparable protection against fungal contamination and the higher germination rates for the wheat grain treated with the *S. canadensis* leaf essential oils suggest that this treatment could be used for grain intended for sowing, and thus especially in organic farming, where the use of synthetic preservatives is not allowed. However, the main obstacle for using essential oils as food preservatives is that they interact with food components and other antimicrobial compounds, and when added in high, active amounts cause negative organoleptic effects (*40*). For these reasons, new antimicrobial blends with components with synergistic effects have been suggested to improve the antimicrobial effects of essential oils. Additional attention should be addressed to different modes of application of essential oils. In contrast to direct treatment of grains, indirect treatment (*i.e.* fumigation) with *Solidago* essential oil would diminish negative organoleptic effects. Previously we showed the potential of this indirect application for the thyme essential oil (*2*). The fumigation with this oil reduced fungal infections of wheat grain while germination rate retained high, suggesting that this method can be used in storage containers to protect grain intended for sowing and food production. When the same fungal species and wheat grain were treated previously with thyme essential oils (*2*, *30*, *49*), these had greater effects on fungal growth and seed germination than for the goldenrod essential oils in the present study. However, the key potential of goldenrod arises from the large biomass availability, from which essential oils and/or extracts can be produced and used to provide more ecologically friendly fungicides.

## CONCLUSIONS

The present study has shown that the invasive Canadian goldenrod (*S. canadensis*) and giant goldenrod (*S. gigantea*) can be used for production of extracts and essential oils that have promising antifungal activities against some of the moulds that are associated with wheat crops. The selection of goldenrod material (*i.e.* in terms of species and tissue) for distillation of these essential oils had no significant impact on the antifungal activities, and therefore whole shoots can be used for their production. Japanese knotweed (*F. japonica*) extracts, however, showed antifungal activities only when prepared from the rhizomes, as those prepared from the leaves did not have any activity. Organic extractions with ethanol and methanol are more effective than aqueous extractions. The results of the study indicate that wheat grain could be soaked or fumigated with *Solidago* essential oils to protect them against fungal infections.
